# Pediatric Hospitalization Due to Trampoline-Related Injuries in the United States During 2019

**DOI:** 10.7759/cureus.74903

**Published:** 2024-12-01

**Authors:** Balagangadhar R Totapally, Ritika Appanagari, Fuad Alkhoury, Keith Meyer

**Affiliations:** 1 Pediatric Critical Care Medicine, Nicklaus Children's Hospital, Miami, USA; 2 Pediatrics, Herbert Wertheim College of Medicine, Miami, USA; 3 Pediatric Surgery, Nicklaus Children's Hospital, Miami, USA

**Keywords:** child, epidemiology, injury, pediatric fractures, trampoline

## Abstract

Background

Despite multiple policy statements from the American Academy of Pediatrics (AAP) and other societies, trampoline is a popular recreational activity among children, leading to multiple injuries. This study aimed to present the hospitalization rate due to trampoline-related injuries in the United States and describe the range of pediatric injuries.

Materials and methods

A cross-sectional analysis was performed utilizing the Kids’ Inpatient Database for 2019. Children from one month to 20 years of age with a discharge diagnosis of trampoline-related injury (TRI) were included. Demographic characteristics and outcome variables were compared between discharges with and without TRI. Age-specific prevalence of hospitalization among all discharges and the US population was analyzed. The chi-square test, Mann-Whitney U test, and regression analyses were used for inferential statistical comparisons.

Results

Out of 2,139,779 discharges in the United States during 2019, TRIs were present in 885, with a prevalence of 41.3 per 100,000 discharges and 10.3 per million US children, accounting for 0.24% of all trauma-associated admissions. Hospitalization with TRI occurred more often over the weekend and during summer months in White male children with private insurance and higher income. Children aged 5-14 years had the highest prevalence of hospitalization. Upper limb fractures were the most common type of injury, followed by lower limb fractures. A severe (intracranial, spinal, or abdominal) injury occurred in 13.2%. Younger children, compared to older children, suffered more intracranial injuries.

Conclusions

Hospitalization with TRIs occurs in 10 per million children annually in the United States. About 13% of these children have serious injuries. Continued advocacy for trampoline safety is essential.

## Introduction

A trampoline is an equipment for acrobatic or gymnastic exercises and recreation. It has been an Olympic sport since 2000 and is popular among children and adolescents [1]. Trampolines consist of a durable fabric sheet connecting springs to a frame. Trampolines are used by families and their children in the backyard for outdoor recreation on summer days or in sports or recreation centers. However, using trampolines can result in injuries. Around half a million trampolines are sold yearly in the United States, and trampoline-related injuries (TRIs) are rising [2]. Between 2008 and 2017, there was a 3.85% increase in trampoline-related pediatric fractures per person-year [3]. However, there was no change in the risk of a trampoline fracture requiring hospitalization during the study period [3]. In the United States, there were an estimated 108,297 emergency room visits for TRIs in children aged 0-17 years in 2022 [4].

Although most injuries involve extremities, permanent neurologic injury happens in one in 200 incidents [5]. Despite multiple policy statements from the American Academy of Pediatrics (AAP, the latest being in 2012) and other societies, trampolining is a popular recreational activity among children, especially at birthday parties and events [1,6,7]. There is a paucity of published literature about hospitalization due to TRIs at a national level. The objective of our study was to evaluate the prevalence of hospitalization due to TRIs in the United States and describe the range of pediatric injuries in those requiring hospitalization using a national pediatric discharge database.

## Materials and methods

We performed a retrospective cross-sectional analysis utilizing the Healthcare Cost and Utilization Project’s (HCUP) Kids’ Inpatient Database (KID) of the Agency for Healthcare Research and Quality (AHRQ) to evaluate the prevalence of hospitalization of children due to TRIs in children in the United States during the year 2019 [8]. According to HCUP, data was sampled from 4,000 US community hospitals (defined as short-term, non-Federal, general, and specialty hospitals, excluding hospital units of other institutions), excluding rehabilitation hospitals for pediatric discharges of all payers, including children covered by Medicaid or private insurance and uninsured. Pediatric discharges (one month to 20 years old or less at admission) were sampled at a rate of 80% [8]. The KID is designed to permit researchers to study a broad range of conditions, specifically rare diagnoses and procedures, related to child health issues. The KID can be used to analyze national trends in healthcare utilization, charges, quality, and outcomes. It excludes data elements that could directly or indirectly identify individuals. It includes 3,998 hospitals and 49 states in the 2019 database. All children from the age of one month to 20 years with the diagnosis of TRI were included in this study. The International Classification of Diseases 10th revision (ICD-10) diagnosis code Y93.44 was used to identify the TRIs. We have used either AHRQ’s Clinical Classifications Software (CCS) codes or the ICD-10 diagnosis and procedure codes to identify associated complications and procedures. The CCS condenses several ICD-10-CM codes into a smaller number of clinically meaningful groups that are more useful for describing the data than individual ICD-10-CM codes.

The age variable was divided into four categories: 29 days-5 years, 6-11 years, 12-18 years, and 19-20 years [9]. Race/ethnicity data was regrouped into White, Black, Hispanic, and other (including Asian/Pacific Islander, Native American, and unspecified). Payor status was grouped as government insurance, private insurance, or other forms of coverage. Median household income (MHI) is categorized into quartiles based on estimated income data from residents’ ZIP codes. These quartiles range from first (lowest) to fourth (highest), indicating the poorest to wealthiest populations, with the income ranges for each quartile varying annually due to updates in ZIP code-based demographic information. The month of admission was grouped into four seasons: spring (March to May), summer (June to August), fall (September to November), and winter (January, February, and December). The location of injury was categorized into home and outside the home. For analysis, a severe injury was defined as having an intracranial injury, spinal cord injury, internal organ injury, or paralysis.

Statistical analysis

Complex sampling and data weighting (for national estimates) were employed for all analyses. Gender, age group, race/ethnicity, MHI of the patient’s zip code, and primary payer were used to describe the study population's demographic characteristics. Demographic characteristics and outcome variables (length of stay and hospital charges) were compared between discharges with and without TRIs among all admissions with trauma diagnoses. Age-specific prevalence of hospitalization among all trauma discharges, all hospital discharges, and the US population was calculated. The prevalence of hospitalization was calculated per 10,000 hospital discharges with trauma diagnosis, 100,000 total hospital discharges, and one million US population. A mid-year age-specific US population was used to measure population prevalence. Chi-square and Mann-Whitney U tests were used for data analysis. A binary regression analysis was conducted to assess the adjusted risk of intracranial injury by incorporating independent variables, age groups, and injury location into the model. The location of injury was documented in 27% of cases. For regression analysis, the missing data for injury location was categorized as unknown. The missing data was not included in other analyses involving the location of injury. Categorical data are summarized using numerical values and percentages with 95% confidence intervals (CIs), while continuous data are depicted using medians and interquartile ranges. Two-by-two analysis and regression results are presented in the form of p-values alongside Odds ratios (ORs) and 95% CIs. P-values less than 0.05 were considered statistically significant. IBM SPSS Statistics for Windows, version 28 (IBM Corp., Armonk, NY), was used for data analysis. All analyses were performed according to the HCUP guidelines, and data with values < 11 patients were not reported. The Western IRB approved this study as exempt. The data used for the study is available for purchase with a license from the HCUP (https://hcup-us.ahrq.gov/).

This article was previously presented as a meeting abstract at the AAP National Conference and Exhibition on October 20-24, 2023, in Washington, DC.

## Results

Out of 2,139,779 pediatric discharges in the United States during 2019, TRIs were present in 885 (817-952) cases, with a prevalence of 41.3 per 100,000 discharges. TRIs were 0.24% (0.22%-0.25%) out of 373,597 (373,490-373,703) admissions with any trauma. The population-based hospitalization rate was 10.3 per million children in the United States. Hospitalization with TRIs occurred more often over the weekend and during spring and summer months in White male children with private insurance who reside in higher-income ZIP codes when compared to other discharges (Table 1). Children aged 5-14 years had the highest prevalence of hospitalization with TRIs. Age-specific hospitalization rates are presented in Table 2.

**Table 1:** Demographic characteristics of children discharged with and without trampoline-related injuries Presented as percentage and 95% confidence intervals. *Chi-square test.

Variables	Trampoline-related injuries (n = 885)	Not trampoline-related injuries (n = 2,139,705)	Significance*
Males	59.9 (56.1-63.6)	43.8 (43.7-43.8)	OR: 1.9 (1.7-2.2); p < 0.001
Age groups			
0-4 years	17.3 (14.6-20.3)	29.4 (29.4-29.5)	<0.001
5-9 years	38.7 (35.1-42.5)	11.8 (11.7-11.8)	
10-14 years	28.0 (24.7-31.5)	14.6 (14.6-14.7)	
15-17 years	10.3 (8.2-12.8)	15.7 (15.6-15.7)	
18-20 years	5.8 (4.2-7.8)	28.5 (28.5-28.6)	
Race			
White	59.0 (55.2-62.7)	45.5 (45.5-45.6)	<0.001
Black	8.6 (6.7-11.0)	17.9 (17.9-18.0)	
Hispanic	20.9 (17.9-24.1)	21.5 (21.5-21.6)	
Others	11.6 (9.3-14.2)	15.0 (15.0-15.0)	
Weekend admission	43.2 (39.4-47.0)	21.5 (21.4-21.6)	OR: 2.8 (2.4-3.2); p < 0.001
Discharge seasons			
Spring	29.7 (26.2-33.3)	25.5 (25.4-25.5)	<0.001
Summer	33.0 (29.5-36.8)	22.8 (22.7-22.9)	
Fall	18.5 (15.6-21.7)	25.1 (25.0-25.2)	
Winter	18.8 (16.0-22.1)	26.6 (26.5-26.7)	
Region of hospital			
Northeast	12.8 (10.5-15.6)	16.9 (16.9-17.0)	<0.001
Midwest	17.4 (14.7-20.4)	22.2 (22.1-22.2)	
South	35.6 (32.0-39.3)	39.4 (39.4-39.5)	
West	34.2 (30.7-38.0)	21.5 (21.4-21.5)	
Median household income			
1st quartile	24.6 (21.5-28.1)	33.5 (33.5-33.6)	<0.001
2nd quartile	23.4 (20.3-26.8)	25.0 (24.9-25.0)	
3rd quartile	26.9 (23.6-30.5)	23.1 (23.1-23.2)	
4th quartile	25.0 (21.9-28.5)	18.4 (18.3-18.4)	
Payor			
Government	41.0 (37.3-44.8)	55.1 (55.1-55.2)	<0.001
Private	48.2 (44.4-52.0)	37.4 (37.3-37.4)	
Others	10.8 (8.6-13.4)	7.5 (7.5-7.6)	

**Table 2:** Frequencies of injuries and procedures in children discharged with trampoline-related injuries during 2019 in the United States Presented as a percentage and 95% confidence intervals. *Serious injuries include intracranial injury, spinal injury, internal organ injury, or paralysis. CNS: Central nervous system (cranial and spinal).

Variables	Trampoline-related injuries (%; 95% CI)
Injuries	
Upper limb fractures	40.1 (36.4-43.9)
Lower limb fractures	31.2 (27.8-34.9)
Serious injuries*	13.2 (10.8-16.0)
Intracranial injury	8.2 (6.3-10.6)
Head/face fractures	7.9 (6.1-10.2)
Other fractures	4.1 (2.9-6.0)
Sprains	3.3 (2.2-5.0)
Internal organ injury	3.0 (2.0-4.7)
Spinal cord injury	2.4 (1.5-3.9)
Femur neck fracture	2.1 (1.3-3.5)
Joint trauma	1.8 (1.0-3.2)
Epilepsy	1.7 (0.9-3.0)
Head/neck open wounds	1.5 (0.8-2.8)
Extremity open wounds	1.2 (0.6-2.5)
Procedures	
Orthopedic operating room procedures	75.4 (72.0-78.6)
CNS therapeutic procedures	2.6 (1.6-4.1)
Intubation and ventilation	1.7 (0.9-3.1)

Injury location was documented in 27% of cases, with half occurring at home and the other half in recreational areas. Upper limb fractures were the most common type of injury, followed by lower limb fractures (Table 3). An intracranial injury occurred in 8.2%, a spinal cord injury in 2.4%, and an internal abdominal injury in 3%, with 13.2% (10.8-16.0) having at least one type of severe injury. Intracranial injury was more common in younger age groups (p < 0.001) and home locations than in sports recreational facilities (OR: 3.3; 95% CI: 1.2-9.4; p = 0.018). In regression analyses that accounted for the child’s age group, it was found that the adjusted odds of intracranial injury were higher at 0-4 years of age compared to 18-20 years of age (aOR: 6.3; 95% CI: 1.04-38.8; p = 0.046).

**Table 3:** In-hospital prevalence and population-based, age-specific hospitalization of trampoline-related injuries during 2019 TRI and total discharges are presented as total number (95% CI). *p < 0.0001 significance level from the chi-square test between age groups and prevalence (hospital or national). In-hospital prevalence is calculated per 100,000 age-specific hospital discharges, and prevalence in the United States is calculated per one million age-specific mid-year US population. TRI: Trampoline-related injuries.

Age groups	TRI discharges (n = 885)	Total hospital discharges (n = 2,139,779)	TRI in-hospital prevalence per 100,000 discharges*	US population	TRI rate per million US population*
29 days through 4 years	153 (125-181)	635,142 (633,616-636,668)	24.0	19,736,000	7.8
5 through 9 years	342 (300-385)	244,823 (243,760-245,885)	139.9	20,212,000	16.9
10 to 14 years	248 (212-283)	324,514 (323,318-325,709)	76.3	20,827,000	11.9
15 to 17 years	91 (69-112)	332,320 (331,111-333,530)	27.3	13,018,000	7.0
18 to 20 years	51 (35-67)	602,981 (601,470-604,492)	8.5	11,851,000	4.3
All ages	885 (817-952)	2,139,779 (2,139,548-2,140,011)	41.3	85,644,000	10.3

Orthopedic operating room procedures were documented in 75.4% (72.0-78.6) and cranio-spinal therapeutic procedures in 2.6% (1.6-4.1) of hospitalized children with TRIs. The median length of stay and hospital charges were two (1.8-2.2) days and $44,484 ($40,287-$48,681), respectively, which is significantly lower than those in the discharges without TRI.

During 2019, there were an estimated 108,542 (69,596-147,489) emergency room visits in children aged 0-20 years with TRIs [10]. Using the National Electronic Injury Surveillance System (NEISS) data, the hospitalization rate was 0.82% for children visiting emergency rooms with TRIs.

## Discussion

The prevalence of hospitalization with TRIs in children was 41.3 per 100,000 discharges and 10.3 per 1000,000 population in the United States in 2019, accounting for 0.24% of all trauma-associated admissions. The NEISS data, between 2008 and 2017, revealed a 3.85% increase in the incidence of trampoline-related pediatric fractures per person-year. The incidence of pediatric trampoline-related fractures increased from 35.3 per 100,000 person-years to 53.0 per 100,000 person-years from 2008 to 2017. However, there was no change in the hospitalization rate due to trampoline fractures [3]. Based on the results from our study and the emergency room visit report from NEISS, the calculated hospitalization rate was 0.82% in those who visited the emergency room for TRIs. To our knowledge, we are describing the demographic and clinical characteristics of the largest cohort of children admitted with TRIs in this report.

Age

The most common age for hospitalization with TRIs in our study was 5-14 years. In agreement with our findings, a report from the NEISS database spanning the years 2002 to 2011 indicated that most trampoline injuries occur within residential settings, with over 90% affecting children, primarily in the age range of 5-14 years [1,11]. The peak age of TRIs may be higher among hospitalized children compared to those who visited the emergency room, as the hospitalization rate is higher in the older age group [12]. Based on our study, advocacy should be directed to possibly limit the age of children who can participate to older children.

Location of injury

In our study, the location of injury was reported only in about a quarter of cases, with 50% of them occurring at home and the rest in sports or recreational centers. In earlier studies, TRIs to children were mostly associated with the use of backyard trampolines in residential settings [2,11,13]. Trampolining made its first appearance at the 2000 Olympic Games in Sydney, Australia. Since then, the number of trampoline parks has increased. The number of businesses in the trampoline parks industry in the United States has grown 5.9% per year on average over the five years between 2018 and 2023 [14]. Trampolining is a form of gymnastics, and trampolinists have greater bone density and higher muscle strength in the lower body than the control group [15]. One of the biggest reasons for the growth of the trampoline park industry is increased awareness of the benefits of physical activity. Trampoline parks offer an enjoyable and dynamic way for individuals to exercise and remain active. An increase in TRIs has accompanied the rapid growth of recreational sports facilities featuring trampolines [16]. Large trampoline centers potentially risk more severe injuries [17]. A systemic review found an increased likelihood of musculoskeletal and/or orthopedic injuries, lower extremity injuries, sprains, and a need for surgery at trampoline centers compared with home trampolines. Children using trampoline centers are more likely to suffer severe trauma and require surgical intervention than children using home trampolines [18]. In a study of 18 trampoline parks operating in Australia and the Middle East, from 2017 to 2019, TRI occurred at a rate of 1.14 injuries per 1000 jumper hours, and the injury rates decreased by 0.72%/month during the study period [19]. In our study, the location of trampoline activity did not affect the risk of intracranial injury. The differences may be due to missing data regarding the location of trampoline activity in our database.

Recreational sports facility-related trampoline injuries occur more often in older age groups, sustain extremity injuries, as shown in our study, and are presented to larger hospitals compared to home trampoline injuries [16]. In a study from Israel, nearly half of the TRIs were fractures, and large trampoline centers posed a potential risk for more serious injuries [17]. A systemic review found an increased likelihood of musculoskeletal and/or orthopedic injuries, lower extremity injuries, sprains, and a need for surgery at trampoline centers compared with home trampolines. Children using trampoline centers are more likely to suffer severe trauma and require surgical intervention than children using home trampolines [18]. Awareness of this information is helpful for anticipatory guidance in pediatric practice and recommending standards for recreational sports facilities to prevent injuries for public health policy.

Seasonality

As trampolining is an outdoor activity, it is not surprising that TRIs in our study were more common during spring and summer and less common in fall and winter. Similarly, in New Zealand, a retrospective review of the trauma database showed that TRIs in children were uncommon in winter, but rose in spring and summer with peak incidence occurring around the beginning of spring daylight saving time each year [20].

Type of injuries

Skeletal injuries are the most common type of injuries from trampoline activity. The type of injury and anatomical location of injury varies with the age of the patient, location of trampoline activity (domestic versus sports/recreation center), and cohort characteristics (emergency room versus hospitalization) [16-18, 21-24]. In our report, we noted that upper limb fractures are the most common followed by lower limb fractures and craniospinal injuries. Our study indicates that younger children are more prone to head injuries. In comparison, older children are more likely to sustain spinal injuries from using a trampoline [23,24]. Fractures of the proximal tibia were particularly common in younger children aged two to five years. Children under five years are at risk for specific proximal tibia fractures known as "trampoline fractures." A child jumping simultaneously with other children is more likely to suffer from a fracture [21].

Craniospinal injuries from trampoline use are uncommon but can be serious. Younger children are more likely to develop a head injury [23,24]. An analysis of the Pennsylvania Trauma Outcome Study database from 2016 to 2018 showed that 9.35% of patients had injuries to the spine and 4.67% had head injuries [5]. Severe injuries are not uncommon in those hospitalized with TRIs, as seen in 13% of our study.

Hospitalization rate

Most TRIs are mild and do not need hospitalization. In a study from South Korea, the risk for hospitalization is higher among children between 13 and 18 years, with injuries to the upper extremities, fractures, falls, and collisions [12]. The hospitalization rate varied in various studies, with a 14.6% admission rate and a 9.5% surgical intervention rate in a study from Singapore [25]. The hospitalization rate in our study at a national level was less than 1% for all children who were seen in an emergency room for TRIs.

Surgery rate

More than 75% of children hospitalized with TRIs required operating room surgical procedures in our study. In the published literature, the surgical rate after TRIs varied depending on the study cohort from 16-62% [13,26,27]. According to indirect meta-analysis methods, children and females had higher surgery rates with full-size and park trampolines than mini-size and home trampolines, adults, and males in TRIs [27].

Prevention

Prevention of TRIs requires a multifaceted approach with education on safe practices, enforcement of safety standards, and improved design of the trampolines. An educational intervention study showed that it can help increase knowledge about safe trampoline practices and increase awareness of injury [28]. The latest guidelines on the use and safety of trampolines were published by the American Academy of Pediatrics in 2012. The AAP reported that the majority of trampoline injuries happen when multiple users are on the mat at the same time. Cervical spine injuries frequently result from falls off the trampoline or attempts at somersaults or flips. The statement strongly discourages the home use of trampolines. Recommendations for enhancing safety in structured training settings are made [6]. The equipment-induced and falling-off injuries, the more severe injuries on traditional trampolines, can be significantly reduced with appropriate trampoline design [29].

Limitations

Studies utilizing administrative databases have several limitations. As an administrative database, the KID relies on data collected from coding and billing entries. Retrospective analyses are susceptible to recall bias and misclassification bias. Chronological relationships cannot be established using the KID database. Additionally, the validity of such studies may be compromised by potential coding errors, including omissions or inaccuracies. The location of trampoline activity was not documented in most cases in our study, which limits the analysis involving injury location. Due to the absence of specific ICD codes, we were unable to identify the particular mechanism of injury, and the location of injury was not reported in a majority of cases. Moreover, some risk factors that may be present but cannot be identified may make such studies misrepresentative.

## Conclusions

Despite guidelines from the AAP and other societies, hundreds of children are hospitalized in the United States each year due to TRIs. While serious injuries from trampolining are uncommon, they can occur. The findings of this study should warn pediatricians and public health officials about the necessity of advocating for trampoline safety and the prevention of serious injuries.
